# Small airway bronchodilator response to different doses of salbutamol in 7-year-old children

**DOI:** 10.1186/s12931-017-0632-8

**Published:** 2017-08-03

**Authors:** Dong Keon Yon, Hye Mi Jee, Eun Kyo Ha, Seung Jin Lee, Young-Ho Jung, Kyung Suk Lee, Man Yong Han

**Affiliations:** Department of Pediatrics, CHA Bundang Medical Center, CHA University School of Medicine, Seongnam, Republic of Korea

**Keywords:** Small airway bronchodilator response, Bronchodilator, Salbutamol, Children

## Abstract

The Global Initiative for Asthma (GINA) guidelines do not specify a bronchodilator range for bronchodilator response (BDR) testing and simply recommend a salbutamol dose of 200 to 400 μg. We determined the oscillometric BDR results of children given low-dose (2 puffs, 200 μg) and standard-dose (4 puffs, 400 μg) salbutamol to compare the small airway responses of healthy controls (defined using criteria based on the guidelines developed at the American Thoracic Society) and exclusion subjects (defined as any child that did not meet the inclusion criteria for healthy controls). The oscillometric reactance of small airways is significantly associated with the dose of salbutamol used for BDR testing in exclusion children. We suggest use of the standard-dose of salbutamol for oscillometric BDR testing.

## Background

The dose of a short-acting beta 2-agonist, such as salbutamol, is associated with the bronchodilator response (BDR) [1]. For evaluation of the BDR, the American Thoracic Society and the European Respiratory Society recommend 4 puffs (400 μg) of salbutamol [2]. The Global Initiative for Asthma (GINA) guidelines do not specify a bronchodilator range for BDR testing, and simply recommend a salbutamol dose of 200 to 400 μg [3]. Most previous studies comparing healthy volunteers with asthmatic subjects [4–8] and epidemiologic studies of children [9, 10] used 2 puffs (200 μg) of salbutamol for oscillometric BDR testing. However, these previous studies used different criteria to define healthy children [9–14]. A dose of 200 to 400 μg salbutamol for conventional BDR testing has been acceptable in clinical practice; on the other hand, oscillometric BDR test results in previous epidemiologic studies [10–14] and patient-control studies [4–8], which used a range of 200 to 300 μg dose salbutamol, are questionable, because oscillometric lung function is more sensitive than conventional spirometry in children [15].

In this study, we determined the oscillometric BDR results of children given low-dose (2 puffs, 200 μg) and standard-dose (4 puffs, 400 μg) salbutamol to compare the small airway responses of healthy controls (defined using criteria based on the guidelines developed at the American Thoracic Society) and exclusion subjects (defined as any child that did not meet the inclusion criteria for healthy controls) [14].

## Methods

We prospectively recruited 248 Korean children who were 7 years-old (167 boys, 81 girls) who participated in the atopy prevention project in the Seongnam Atopy Prevention program (SAP 2016) between January 2016 and December 2016. The present study was designed as a cross-sectional and general population-based study including 11 randomly selected elementary schools from Seongnam city, Gyeonggi province, Republic of Korea for the prevalence of allergic diseases in children. All parents or caregivers signed written informed consent documents, and the study protocol was approved by the appropriate Institutional Review Board of CHA University.

We performed the oscillometric [16] and spirometry [15] BDR tests according to current guidelines. The pulmonary function tool was the impulse oscillometry (IOS) system from Jaeger Company (Würzburg, Germany). Each subject was given salbutamol (2 or 4 puffs), using a randomized and physician-blinded method, prior to IOS BDR testing. We performed computerized randomization by generated random number series assigned to the low-dose and standard-dose salbutamol groups. The absolute changes and relative changes with respect to baseline respiratory function in reactance at 5 Hz (Xrs5) and 10 Hz (Xrs10), resistance at 5 Hz (Rrs5) and 10 Hz (Rrs10), reactance area (AX), and difference of Rrs5 and Rrs20 (Rrs20–5) were determined.

We reviewed the questionnaires to identify factors related to pulmonary function. A healthy child (*n* = 168) was one who had [14]: 1) no history of acute or past chronic disease, major respiratory disease, or thoracic surgery (excluded: *n* = 20); 2) no systemic disease which could influence the respiratory tract (excluded: *n* = 0); 3) no exposure to second-hand smoke (excluded: *n* = 22); 4) normal body mass index (2 < BMI *z*-score < −2) (excluded: *n* = 23); 5) no upper respiratory tract infection in the previous 1 month (excluded: *n* = 5); 6) gestational age of at least 37 weeks, birth weight of at least 2.5 kg, and no history of transient respiratory problems during the neonatal period (excluded: *n* = 43); 7) predicted forced expiratory volume in 1 s (FEV_1_) greater than 80% (excluded: *n* = 9). Data was analyzed using SPSS version 23.0 (IBM Co, Armonk, NY, USA). A *P* value of <0.05 was considered statistically significant.

## Results

Subjects were categorized as having received 2 puffs (83 healthy controls and 41 exclusion subjects) or 4 puffs (85 healthy controls and 39 exclusion subjects) of salbutamol. The 168 healthy volunteers and the 80 exclusion children had no significant differences in anthropometric and spirometric lung function data (mean age: 7.2 ± 1.4 years versus 7.1 ± 1.6, *p* = 0.648; mean height: 1.24 ± 0.09 m versus 1.20 ± 0.08, *p* = 0.652; mean BMI *z*-score: −0.05 ± 0.85 versus − 0.08 ± 1.57, *p* = 0.872; mean FEV_1_ z-score: 0.31 ± 1.18 versus 0.06 ± 1.30, *p* = 0.101; percentage of males: 55.4% versus 52.2%, *p* = 0.609).

Comparison of the oscillometric BDR data of healthy subjects in the low-dose and standard-dose groups indicated no significant absolute or relative differences in Xrs5, Xrs10, Rrs5, Rrs10, AX, and Rrs20–5 (Table 1). However, the exclusion subjects in the low-dose and standard-dose groups had significant differences in relative Xrs5 (baseline: −19.4 ± 14.4% versus − 26.0 ± 12.6, *p* = 0.032), Xrs10 (baseline: −26.7 ± 50.6% versus − 47.1 ± 15.4, *p* = 0.017), and AX (baseline: −28.4 ± 36.8% versus − 43.7 ± 12.2, *p* = 0.015) and in absolute Xrs10 (0.06 versus 0.08, *p* = 0.043) (Table 1).

**Table 1:** Impulse oscillation measurements of bronchodilator response following low-dose (2 puffs, 200 μg) and standard-dose (4 puffs, 400 μg) salbutamol in healthy controls and exclusion subjects (*n* = 248)

	Healthy controls (*n* = 168)			Exclusion subjects (*n* = 80)		
	Low-dose salbutamol (*n* = 83)	Standard-dose salbutamol (*n* = 85)	*P* value	Low-dose salbutamol (*n* = 41)	Standard-dose salbutamol (*n* = 39)	*P* value
% change in FEV_1_	5.3 (5.7)	5.4 (5.7)	0.984	9.2 (8.4)	7.4 (7.1)	0.317
Xrs5						
Δ abs, hPa s L^−1^ (SD)	0.05 (0.04)	0.05 (0.05)	0.984	0.06 (0.06)	0.07 (0.04)	0.169
Δ relative, % of baseline (SD)	−18.2 (11.3)	−18.3 (19.4)	0.986	**−19.4 (14.4)**	**−26.0 (12.6)**	**0.032**
Rrs5						
Δ abs, hPa s L^−1^ (SD)	−0.10 (0.06)	−0.09 (0.06)	0.628	−0.10 (0.08)	−0.12 (0.06)	0.251
Δ relative, % of baseline (SD)	−14.5 (8.4)	−14.5 (9.3)	0.694	−15.7 (10.8)	−18.7 (8.2)	0.179
Xrs10						
Δ abs, hPa s L^−1^ (SD)	0.06 (0.04)	0.06 (0.04)	0.765	**0.06 (0.06)**	**0.08 (0.04)**	**0.043**
Δ relative, % of baseline (SD)	−34.4 (19.3)	−34.8 (22.2)	0.897	**−26.7 (50.6)**	**−47.1 (15.4)**	**0.017**
Rrs10						
Δ abs, hPa s L^−1^ (SD)	−0.06 (0.04)	−0.05 (0.05)	0.609	−0.05 (0.04)	−0.07 (0.04)	0.172
Δ relative, % of baseline (SD)	−10.8 (7.9)	−10.3 (8.2)	0.679	−10.6 (7.6)	−13.1 (6.8)	0.126
AX						
Δ abs, hPa L^−1^ (SD)	−0.61 (0.37)	−0.58 (0.36)	0.615	−0.63 (0.58)	−0.81 (0.36)	0.091
Δ relative, % of baseline (SD)	−32.3 (15.9)	−32.1 (18.1)	0.950	**−28.4 (36.8)**	**−43.7 (12.2)**	**0.015**
Rrs20–5						
Δ abs, hPa s L^−1^ (SD)	−0.06 (0.08)	−0.08 (0.05)	0.289	−0.06 (0.08)	−0.08 (0.05)	0.294
Δrelative, % of baseline (SD)	−12.3 (75.6)	−30.3 (12.7)	0.147	−12.3 (75.6)	−30.3 (12.7)	0.147

*Δabs* absolute change from the initial value, *Δrelative* relative change from the initial value, *% change in FEV*_*1*_ percentage change in FEV_1_ over baseline, *Xrs5* reactance at 5 Hz, *Xrs10* reactance at 10 Hz, *Rrs5* resistance at 5 Hz, *Rrs10* resistance at 10 Hz, *AX* reactance area, *Rrs20–5* difference of Rrs5 and Rrs20

Numbers in bold indicate a significant difference between the low-dose and standard-dose salbutamol groups (*P* < 0.05)

## Discussion

Our results show that the oscillometric BDR data of healthy controls were similar for children given standard and low doses of salbutamol. In fact, epidemiological studies accept standard BDR reference values based on oscillometric BDR data from low-dose salbutamol in healthy volunteers [9–13]. However, previous researchers have used different criteria to define “healthy control” such as the following: no history of asthma, cystic fibrosis, neonatal chronic lung disease, or respiratory infection in the 2 weeks prior to study onset (Thamrin et al. criteria) [10, 11]; and the Thamrin et al. criteria in addition to no preterm and low birth weight infants (>36 weeks gestational age and >2.5 kg of birth weight) who had not received oxygen at birth [12, 13]; in addition to no exposure to second-hand smoke and normal body mass index such as criteria of the American Thoracic Society [14]. We used a more stringent set of criteria for healthy children than the criteria of the American Thoracic Society because we added an additional criteria of FEV_1_ [14]. Although the healthy group had similar low-dose and standard-dose BDR data, the exclusion group had significantly different BDR data following low-dose and standard-dose salbutamol. Thus, the use of oscillometric BDR data should be carefully considered in epidemiologic studies of children.

Our results show that the measured reactance of small airways in exclusion children depended on the dose of salbutamol used for BDR testing. Interestingly, all previous pediatric oscillometric patient-control studies only used low-dose salbutamol for BDR testing [4–8]. As far as we know, this study is the first to analyze oscillometric small airway hyper-responsiveness in children using low and standard doses of a bronchodilator. The BDR results following low-dose salbutamol may not accurately reflect the reactance of small airway dysfunction. Many researchers have shown that resistance is a more sensitive measure of airway caliber than reactance in children with asthma [6, 8–10]. Our study suggests that use of low-dose bronchodilator may make it difficult to predict the reversibility of reactance in oscillometric BDR testing.

There is limited clinical IOS results in the exclusion group. In particular, the exclusion group presents various characteristics with children representing different phenotype. It was difficult to further divide the exclusion group for homogenous phenotype due to insufficient sample size. We expect further research as a homogeneous phenotype group such as exclusively asthmatic patients compared to healthy group.

## Conclusions

Use of low-dose and standard-dose salbutamol for oscillometric BDR testing yielded similar results in healthy children, although our criteria for “healthy” were more stringent than those of other studies. The oscillometric reactance of small airways is significantly associated with the dose of salbutamol used for BDR testing in exclusion children. For further examination of reactance, we suggest use of the standard dose of salbutamol for oscillometric BDR testing. Since there is no accurate BDR guideline for other pulmonary function tests, many researchers extended the GINA guideline for 200-400μg of salbutamol for BDR to the IOS [4–13]. It is also necessary for the GINA guidelines to specify the amount of salbutamol inhaler to be used for BDR testing to the IOS.

## Abbreviations

AX: Reactance area
BDR: Bronchodilator response
BMI: Body mass index
FEV_1_: Forced expiratory volume in 1 s
GINA: Global Initiative for Asthma
IOS: Impulse oscillometry
Rrs10: Resistance at 10 Hz
Rrs20–5: Difference of Rrs5 and Rrs20
Rrs5: Resistance at 5 Hz
Xrs10: Reactance at 10 Hz
Xrs5: Reactance at 5 Hz

## References

[1] Nelson HS, Spector SL, Whitsett TL, George RB, Dwek JH (1983). The bronchodilator response to inhalation of increasing doses of aerosolized albuterol. J Allergy Clin Immunol.

[2] Pellegrino R, Viegi G, Brusasco V, Crapo RO, Burgos F, Casaburi R, Coates A, van der Grinten CPM, Gustafsson P, Hankinson J (2005). Interpretative strategies for lung function tests. Eur Respir J.

[3] Global Initiative for Asthma (2016). Global strategy for asthma management and prevention.

[4] Hellinckx J, De Boeck K, Bande-Knops J, van der Poel M, Demedts M (1998). Bronchodilator response in 3-6.5 years old healthy and stable asthmatic children. Eur Respir J.

[5] Ortiz G, Menendez R (2002). The effects of inhaled albuterol and salmeterol in 2- to 5-year-old asthmatic children as measured by impulse oscillometry. J Asthma.

[6] Lall CA, Cheng N, Hernandez P, Pianosi PT, Dali Z, Abouzied A, Maksym GN (2007). Airway resistance variability and response to bronchodilator in children with asthma. Eur Respir J.

[7] Oostveen E, Dom S, Desager K, Hagendorens M, De Backer W, Weyler J (2010). Lung function and bronchodilator response in 4-year-old children with different wheezing phenotypes. Eur Respir J.

[8] Vu LT, Demoulin B, Nguyen MT, Nguyen YT, Marchal F (2010). Respiratory impedance and response to salbutamol in asthmatic Vietnamese children. Pediatr Pulmonol.

[9] Malmberg LP, Pelkonen A, Poussa T, Pohianpalo A, Haahtela T, Turpeinen M (2002). Determinants of respiratory system input impedance and bronchodilator response in healthy Finnish preschool children. Clin Physiol Funct Imaging.

[10] Thamrin C, Gangell CL, Udomittipong K, Kusel MM, Patterson H, Fukushima T, Schultz A, Hall GL, Stick SM, Sly PD (2007). Assessment of bronchodilator responsiveness in preschool children using forced oscillations. Thorax.

[11] Vu LT, Demoulin B, Nguyen YT, Nguyen MT, Marchal F (2008). Respiratory impedance and response to salbutamol in healthy Vietnamese children. Pediatr Pulmonol.

[12] Calogero C, Parri N, Baccini A, Cuomo B, Palumbo M, Novembre E, Morello P, Azzari C, De Martino M, Sly P (2010). Respiratory impedance and bronchodilator response in healthy Italian preschool children. Pediat Pulmonol.

[13] Calogero C, Simpson SJ, Lombardi E, Parri N, Cuomo B, Palumbo M, de Martino M, Shackleton C, Verheggen M, Gavidia T (2013). Respiratory impedance and bronchodilator responsiveness in healthy children aged 2–13 years. Pediatr Pulmonol.

[14] Stocks J, Quanjer PH (1995). Reference values for residual volume, functional residual capacity and total lung capacity. ATS workshop on lung volume measurements. Official statement of the European Respiratory Society. Eur Respir J.

[15] Shin YH, Jang SJ, Yoon JW, Jee HM, Choi SH, Yum HY, Han MY (2012). Oscillometric and spirometric bronchodilator response in preschool children with and without asthma. Can Respir J.

[16] Shin YH, Yoon JW, Choi SH, Baek JH, Kim HY, Jee HM, Yum HY, Han MY (2013). Use of impulse oscillometry system in assessment of asthma severity for preschool children. J Asthma.

